# Low Dose X-Ray Sources and High Quantum Efficiency Sensors: The Next Challenge in Dental Digital Imaging?

**DOI:** 10.1155/2014/543524

**Published:** 2014-12-10

**Authors:** Arnav R. Mistry, Daniel Uzbelger Feldman, Jie Yang, Eric Ryterski

**Affiliations:** ^1^Department of Endodontology, Temple University Kornberg School of Dentistry, 3223 North Broad Street, Philadelphia, PA 19140, USA; ^2^Oral and Maxillofacial Radiology Department, Temple University Kornberg School of Dentistry, 3223 North Broad Street, Philadelphia, PA 19140, USA; ^3^E3 Medical, Inc., 941 Garfield Avenue, Louisville, CO 80027, USA

## Abstract

*Objective(s)*. The major challenge encountered to decrease the milliamperes (mA) level in X-ray imaging systems is the quantum noise phenomena. This investigation evaluated dose exposure and image resolution of a low dose X-ray imaging (LDXI) prototype comprising a low mA X-ray source and a novel microlens-based sensor relative to current imaging technologies. *Study Design*. A LDXI in static (group 1) and dynamic (group 2) modes was compared to medical fluoroscopy (group 3), digital intraoral radiography (group 4), and CBCT scan (group 5) using a dental phantom. *Results*. The Mann-Whitney test showed no statistical significance (*α* = 0.01) in dose exposure between groups 1 and 3 and 1 and 4 and timing exposure (seconds) between groups 1 and 5 and 2 and 3. Image resolution test showed group 1 > group 4 > group 2 > group 3 > group 5. *Conclusions*. The LDXI proved the concept for obtaining a high definition image resolution for static and dynamic radiography at lower or similar dose exposure and smaller pixel size, respectively, when compared to current imaging technologies. Lower mA at the X-ray source and high QE at the detector level principles with microlens could be applied to current imaging technologies to considerably reduce dose exposure without compromising image resolution in the near future.

## 1. Introduction

With all other technical factors (e.g., kilovolts, distance, time, etc.) held constant, patient radiation dose is directly proportional to the milliamperes (mA). A 50% reduction in mA would result in a decrease in radiation dose by 50% [1]. Previously, the mA range has not been taken into consideration in any attempt to reduce radiation dose to which dental patients are being exposed [2–6]. The sensitivity of a digital sensor is measured at a constant wavelength in nanometers (nm) on the basis of the detective quantum efficiency (DQE). This value is used primarily to describe imaging detectors in optical imaging and medical radiography [7]. The quantum efficiency (QE) is the ratio of impinging photons on a pixel to the number of collected electrons. The QE of the pixel is equal to the QE of the complementary metal oxide semiconductor (CMOS) photodiode multiplied for the fill factor of the pixel [8]. Fluoroscopy is a dynamic X-ray or X-ray movie showing images of video frame rates produced by a low mA X-ray source and image intensification at the detector level [9]. An image intensifier unit is capable of multiplying 1,000 to 20,000 times, electron-by-electron, of the produced image, therefore increasing the system QE while allowing dose reduction [10, 11]. Unfortunately, image intensifier units and direct radiography large flat panel detectors have heretofore been too bulky to be used inside the mouth as well as being expensive for a dental setting [12–14]. Another disadvantage of intensifiers is image distortion [15]. With regard to visualization of a stent created from 50 microns (*μ*m) diameter wires in flat panel X-ray fluoroscopy, for the idealized direct detector, the 100 *μ*m pixel size resulted in maximum measured contrast sensitivity. For an idealized indirect detector, with a scintillating layer, the maximal measured contrast sensitivity was obtained at 200 *μ*m pixel size [16].

**Figure 1:**
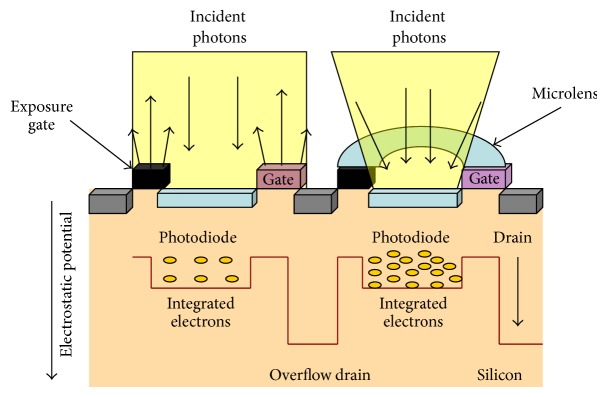
CMOS sensor and microlens (right side) architecture scheme.

The major challenge encountered to decrease the mA level in X-ray imaging systems is the quantum noise phenomena. In electronic imaging systems such as fluoroscopy or intraoral radiography digital sensors, we find three principal sources of noise. The first and most relevant arises from quantum statistics, in which the discrete nature of the radiographic signal (which often is photon-starved) introduces uncertainty into the image. The second is electronic noise which is generated in the detector or detector electronics. The third is quantization error that occurs in digital electronic imaging systems when the signal is digitized. For a quantum statistics noise limited system, if the number of photons used is quadrupled, the noise in the resultant image should be halved  [17–20]. Not only X-ray systems but also digital cameras are noise limited and quantum limited. Randomly spaced speckles, called noise, can appear in digital images. Noise is similar to grain that appears in photos taken with traditional cameras using high International Organization of Standardization (ISO) films. Noise increases in photos taken with a digital camera using a high ISO number. The higher ISO number leads to more noise. When noise is present, image detail and clarity are reduced, sometimes significantly. The ISO level indicates the film and digital camera's sensitivity to light. According to the ANSI/ISO classification, a dental film with raw speed of ISO 29–56 would be classified as E speed, while one with speed of ISO 57–112 would be classified as F speed [21]. Photographic film typically has a QE of much less than 10% [22]. Current digital cameras have improved their ISO settings which can achieve up to ISO 204,800 [23–25]. To improve the sensitivity or QE of front illuminated charged couple device (CCD) and CMOS image sensors without increasing their pixel size, digital cameras manufacturers apply a thin (0.5–1.0 mm thickness) and inexpensive microlens array to the sensors to reach high ISO levels [26]. The microlens principle was invented in the 17th century where Hooke and Van Leeuwenhoek developed techniques to make small glass lenses for use with their microscopes [27]. The microlens collects and focuses light that would have otherwise fallen onto the nonsensitive areas of the sensor chip, improving the QE significantly (Figure 1) [28].

Attempts for improving digital sensors QE without compromising the system's noise have been made through the introduction of back illuminated and electron multiplied CCD and CMOS image sensors. Their major drawbacks are complicated manufacturing processes and elevated cost [29, 30]. As a result, the most common used image sensor in dentistry is the front illuminated type [31].

Consequently, a lower milliamperes (mA) setting at the X-ray source and the use of front illuminated sensors with microlens or back illuminated sensors at the detector level for an increased QE that reduces the required radiation dose and sensor pixel size without impacting image quality should have a dramatic positive impact on dental radiology and oral diagnosis. The purpose of this investigation was to prove the concept of radiation exposure reduction and dynamic fluoroscopy feasibility in dentistry without compromising image quality by testing a low-dose X-ray imaging (LDXI) prototype comprising a low-dose X-ray source and a high QE front illuminated sensor with microlens and comparing it to standard of care in terms of dose exposure in milligrays (mGy) and image resolution in lines per millimeters (lp/mm) [6].

## 2. Materials and Methods

**Figure 2:**
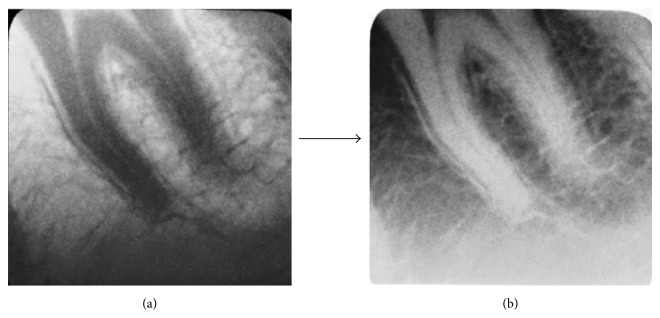
(a) Molar tooth raw positive image obtained at 0.2 mA and 1/30 camera shutter (0.03 seconds) with collimation and (b) negative image.

The Temple University Environmental Health and Radiation Safety Institutional Department approved this study. A LDXI prototype (Real Time Imaging Technologies, LLC, Cleveland, OH) was used. The LDXI was comprised of a 35–80 kilovolt peak (kVp), 0.1–0.5 mA X-ray source (9.95′ L × 5.27′ W × 5.35′ H) (SourceRay, Bohemia, NY), and a 8′ rectangular collimator (Margraf Dental, Jenkintown, PA) and an X-ray detector utilizing a CMOS front illuminated sensor (EOS 5D Mark III, Canon, Japan) having 36 × 24 mm effective area, 6.25 × 6.25 um pixel size, 22.3 megapixels resolution, and 49% [32] QE with microlens and capable of performing up to 30 frames per second (fps) for the dynamic video mode. The CMOS sensor modular transfer function (MTF) was >30% at the 18 lp/mm range. Upon low-pass filter and sensor removal, the scintillator/fiber optic plate (AppliedScintech, UK) was coupled at the sensor [33, 34] on top of the microlens [35] with optic glue (BEW Engineering, Ketsch, Germany).  The scintillator/fiber optic plate measured 20.8 × 20.8 mm and was comprised of a 6 um columnar cesium iodide with thallium (CsI : TI) coating with a MTF curve showing and ultimate resolution >18 lp/mm at 60 kVp with 98% attenuation at 70 kVp. Camera software (EOS Utility Ink, Japan) and a kid's watch (Disney Store, Orlando, FL) were used in order to establish and calibrate sensor's ISO settings. Images obtained were raw positive still shots and video. Raw images (A) were converted to negative radiographic images (B) through a software and then cropped (Microsoft Digital Suite 2006 Editor, Microsoft, Redmond, WA) (Figure 2).

**Figure 3:**
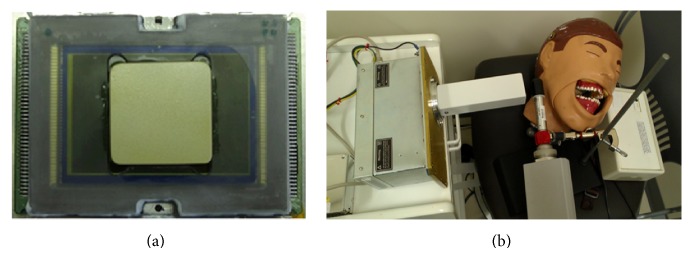
(a) Dental size sensor with microlens coupled with scintillator/FOP (front view) and (b) LDXI prototype testing on the DXTTR phantom at 0.2 mA and 80 kVp.

Ionization chambers Radcal 9010 (Radcal Corp., Monrovia, CA) and RaySafe Xi (RaySafe, Hopkinton, MA) were used to measure the dose exposure in mGy (obtained through rad formula conversion) received by a patient phantom (DXTTR, RINN, Elgin, IL) in all groups and sixteen dosimeters (Landauer, Inc., Glenwood, IL) were utilized for obtaining the dose equivalent in millisievert (mSv) (obtained through rem formula conversion) received by the operator simulated distance at 30 cm for LDXI and fluoroscopy (Figure 3).

ISO settings were established at ISO 5,000 for group 1 and ISO 12,800 for group 2 and image acquisition was made at 1/30 shutter (0.03 seconds). Group 1 was exposed to the LDXI prototype (Real Time Imaging Technologies, LLC, Cleveland, OH) at 0.2 mA and 80 kVp in static mode during 10 intervals from 0 to 27 continuous seconds with a 6.25 × 6.25 um pixel size sensor and 49% QE. Group 2 was exposed to the LDXI prototype at 0.2 mA and 80 kVp in the dynamic video mode during 31 intervals from 0 to 300 seconds with a 6.25 × 6.25 um pixel size sensor and 49% QE. Group 3 was exposed to medical fluoroscopy (GE C-arm OEC 9800, Cleveland, OH) at 0.038 mA and 55 kVp in 31 intervals from 0 to 300 seconds with a 12.8 × 12.8 um pixel size sensor and a 9′ image intensifier with 65% QE at 550 nm [36]. Group 4 was exposed to digital intraoral radiography (Gendex GX-770, Hatfield, PA/Planmeca Dixi 2 v3, Roselle, IL) at 7 mA and 70 kVp in 29 intervals from 0 to 1.65 seconds with a 19 × 19 um pixel size sensor. Group 5 was exposed to CBCT Scan (iCat Imaging Sciences International Inc., Hatfield, PA) at 5 mA and 120 kVp from 0 to 26.9 seconds in 28 intervals and several modalities with a 125 um voxel size sensor. Six thermoluminescent Luxel and ten optical stimulated luminescence technology Nanodots dosimeters (Landauer, Inc., Glenwood, IL) were used at the patient phantom, area monitor, and positive and negative controls for the LDXI. After research completion, all the dosimeters were mailed back to Landauer for analysis purposes. Image resolution for the LDXI was measured with a resolution test pattern (Fluke Corp., Cleveland, OH) in lp/mm. An endodontic file size 10 (Dentsply, Maillefer, York, PA) was placed within phantom's tooth number 27 at the radiographic apex for intraoral imaging subjective resolution assessment purposes in which two endodontists and one oral and maxillofacial radiologist from the institution were asked to confirm tooth's working length [6]. Dose exposure measurements and image resolution were calculated for all groups and dosimeters were analyzed.

## 3. Results

**Figure 4:**
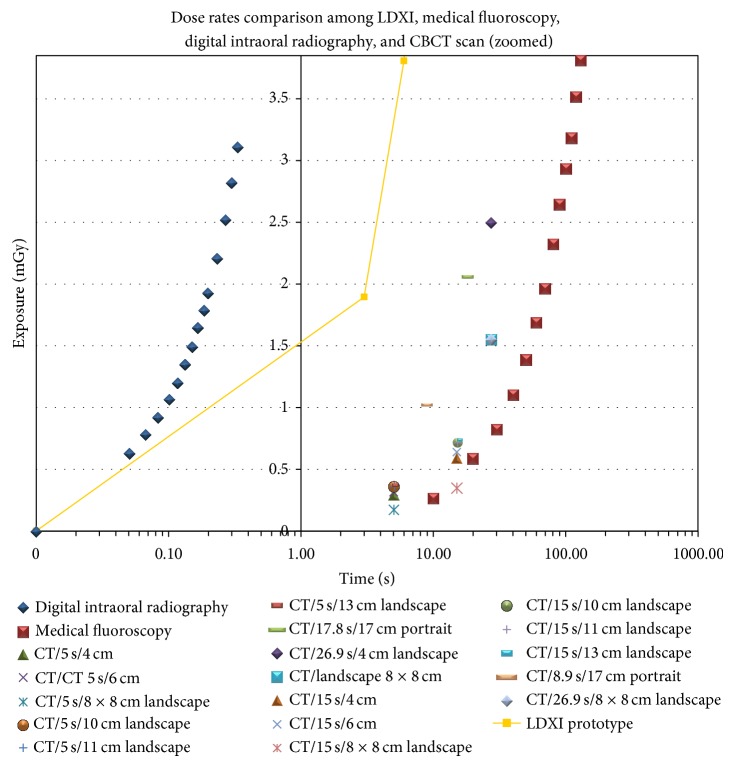
Radiation and timing exposures comparison amongst all groups.

The Mann-Whitney test showed no statistical significance (*α* = 0.01) in dose exposure (mGy) between groups 1 and 3 and 1 and 4 and timing exposure (seconds) between groups 1 and 5 and 2 and 3 (Figure 4).

Dosimeters for the LDXI operator simulated distance and controls did not register significant dose equivalent (mSv).

Image resolution test showed LDXI 1 > digital intraoral radiography > LDXI 2 > medical fluoroscopy > CBCT scan (Table 1).

**Figure 5:**
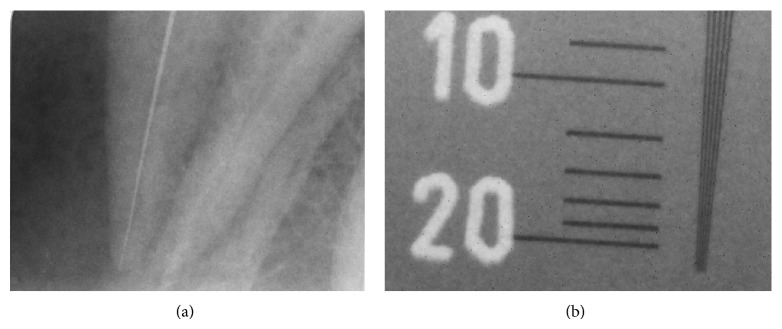
Group 1 LDXI static: (a) endodontic file number 10 at working length and (b) 18 lp/mm.

File size 10 was observed at the working length as confirmed by two endodontists and one oral and maxillofacial radiologist (Figure 5).

## 4. Discussion

**Figure 6:**
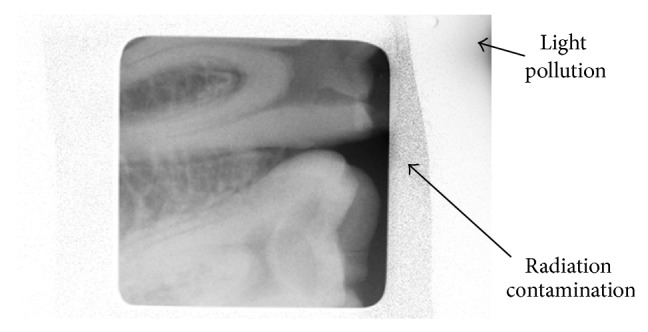
Preventable artifacts obtained in images.

Technical advances enable cameras to better capture the drama of low-light photography/video. As a rule, bigger is indeed better when it comes to the overall dimensions of a sensor because a bigger sensor provides not only more pixels, but also bigger pixels, which more efficiently gather light. However, of all the improvements in the imaging field, perhaps the most notable are not the increases in sensor size but the innovations in increasing a sensor's ability to gather light in low-light situations and to record a wider range of light. Much improved sensors with microlens now allow one to obtain detailed low-light images by allowing shots at high ISO levels that previously generated unflattering digital “noise” or graininess in an image at smaller pixel sizes. From an image resolution dental perspective, studies have demonstrated that spatial resolution affects bone loss and caries diagnosis in dentistry. A research undertaken to determine the effect of X-ray beam alignment and spatial resolution on quantification of alveolar bone using radiometric techniques concluded that 50 um pixel spatial resolution is apparently superior to 200 um pixel images if radiometric data is to be evaluated [37]. Another study determined that spatial resolution affected radiometric analyses aimed at detecting progressive enamel loss. Cumulative percent histograms shifts associated with the smaller 59 um pixels accounted for 68% of the variation in weights caused by enamel reduction, whereas the shifts associated with the larger 200 um pixels accounted for 50%. The results indicated that pixel size does affect radiometric determinations of enamel reduction [38, 39]. In summary, the smaller the pixel size and the more pixels are arranged in a sensor, the better the quality of the image that is captured. In this study, microlens also allows decreasing the sensor's pixel size (6.25 × 6.25 um), therefore increasing image spatial resolution as compared to the larger pixel size of current sensors used in digital intraoral radiography (19 × 19 um), CBCT scans (125 um voxel), and medical fluoroscopy (12.5 × 12.5 um for image intensifiers and 100 × 100 um for flat panel detectors). As compared to similar front illuminated intraoral sensors used in dentistry (DQE 3% CCD and DQE 18% CMOS) [40, 41] the LDXI prototype has used a large size (35 mm), full frame, and front illuminated CMOS sensor with a thin microlens array for improved DQE (49%) directly coupled to the scintillator/fiber optic plate, capable of acquiring low mA images at ISO 5,000 and ISO 12,800 as well as in dynamic video frame rates. From a radiation exposure perspective, we found that 3.05 and 3.95 seconds of LDXI are comparable to one single shot of 0.2 seconds of digital intraoral radiography and 26.9 seconds 4 cm landscape single area CBCT scan, respectively. As a result, the LDXI used for 0.2 seconds would reduce 93.4% dose exposure as compared to digital intraoral radiography used for 0.2 seconds. For a conventional root canal requiring a pre-op, working length, master cone, partial condensation, and final X-ray, the LDXI prototype could be used during 15 seconds without producing more dose exposure due to the addition of microlens for increased QE as compared to digital intraoral radiography. As a result, lower mA settings (up to 0.2 mA) as compared to digital intraoral radiography (7 mA) could be captured at 18 lp/mm and 10 lp/mm image resolution for the static and dynamic modalities, respectively [6]. In addition, an endodontic file size 10 was observed at the working length at the phantom as confirmed by two endodontists and one oral and maxillofacial radiologist from the institution (Figure 5). In addition to root canals, other clinical applications for low dose X-ray sources and high QE image sensors in the static mode would be full mouth X-rays, pre- and postradiographs, bitewings, and panoramic, cephalometric, and CBCT scans for oral diagnosis while the dynamic mode would allow the introduction of fluoroscopy in dentistry for dental implant placement, temporomandibular joint analysis, maxillofacial surgeries, and postplacement. Since the scintillator/fiber optic plate was coupled in the central area of the sensor, the effect of X-rays on the surrounding, residual, and uncovered area caused significant noise causing black spots at the image. In addition, despite the fact that sensor's housing was internally masked against light, light pollution was observed on one corner (Figure 6).

These artifacts should be avoided in future studies by providing complete sensor shielding and a 100% light proof housing. In addition to further evaluations with higher QE (>65% at 550 nm) new generation front illuminated sensors with microlens, the authors recommend testing a back illuminated sensor due to its increased QE as compared to the front illuminated sensor used in this study [14, 35]. Finally, we propose testing the next generation LDXI using head and neck anthropomorphic phantoms following Sections C and D of the FDA guidance for Solid State X-Ray Imaging Devices (MTF, detective quantum efficiency, and signal-to-noise ratio) and compare it to digital intraoral radiography, medical fluoroscopy, and CBCT scan imaging devices.

## 5. Conclusions

The LDXI proved the concept for obtaining a high definition image resolution for static and dynamic radiography at lower or similar dose exposure and smaller pixel size, respectively, when compared to traditional imaging devices. Lower mA at the X-ray source and high QE at the detector level principles with microlens could be applied not only to digital intraoral radiography and dental fluoroscopy but also to panoramic, cephalometric, and CBCT scans devices to considerably reduce X-ray source dose exposure as well as sensor pixel size and more research is recommended to demonstrate this further.

## Figures and Tables

**Table 1:** mA, kVp, pixel size settings, and resolution outcome comparison of different devices.

Device type	mA	kVp	Pixel/voxel size (um)	Resolution (lp/mm)
LDXI 1 (static)	0.2	80	6.25 × 6.25	18

LDXI 2 (dynamic)	0.2	80	6.25 × 6.25	10

Medical fluoroscopy (image intensified)	0.038	55	12.8 × 12.8	1.75

Digital intraoral radiography	7	70	19 × 19	16

CBCT scan	5	120	125	1.6

## Diclosure

The paper, or any part of it, has not been submitted or published and will not be submitted elsewhere for publication while being considered by the journal. The research has been shown as a poster presentation at Temple University Kornberg School of Dentistry Research Day and has been presented at the 64th AAOMR Annual Session as an abstract and as part of the 2013 William H. Rollins Award Lecture.
